# Comparative Platelet Releasate Proteomic Profiling of Acute Coronary Syndrome versus Stable Coronary Artery Disease

**DOI:** 10.3389/fcvm.2020.00101

**Published:** 2020-06-24

**Authors:** Patricia B. Maguire, Martin E. Parsons, Paulina B. Szklanna, Monika Zdanyte, Patrick Münzer, Madhumita Chatterjee, Kieran Wynne, Dominik Rath, Shane P. Comer, Melanie Hayden, Fionnuala Ní Áinle, Meinrad Gawaz

**Affiliations:** ^1^Conway SPHERE Research Group, Conway Institute, University College Dublin, Dublin, Ireland; ^2^School of Biomolecular and Biomedical Science, University College Dublin, Dublin, Ireland; ^3^UCD Institute for Discovery, University College Dublin, Dublin, Ireland; ^4^Irish Centre for Vascular Biology, Dublin, Ireland; ^5^Universitätsklinikum Tübingen, Medizinische Klinik III, Kardiologie und Kreislauferkrankungen, Tübingen, Germany; ^6^Proteomics Core, Conway Institute, University College Dublin, Dublin, Ireland; ^7^School of Medicine, University College Dublin, Dublin, Ireland; ^8^Department of Haematology, Rotunda Hospital, Dublin, Ireland; ^9^Department of Haematology, Mater Misericordiae University Hospital, Dublin, Ireland

**Keywords:** platelets, platelet releasate, proteomics, mass spectrometry, acute coronary syndrome, stable coronary artery disease

## Abstract

Upon activation, platelets release a host of soluble and vesicular signals, collectively termed the “platelet releasate” (PR). The contents of this PR play a significant role in haemostasis, inflammation, and pathologic *sequelae*. Despite this, proteomic studies investigating the PR in coronary artery disease have not been performed. Here, we undertook a comparative label-free quantitative (LFQ) proteomic profiling of the 1 U/ml thrombin-induced PR from 13 acute coronary syndrome vs. 14 stable angina pectoris patients using a tandem mass spectrometry approach. Data are available via ProteomeXchange with identifier PXD009356. 318 PR proteins were identified across both cohorts with 9 proteins found to be differentially released, including tetranectin (CLEC3B), protein disulfide-isomerase-A3 (PDIA3), coagulation factor V (F5), and fibronectin (FN1). Strikingly, these 9 differential proteins were all associated with the gene ontology cellular component term “extracellular vesicle” and reduced levels of EVs were detected in the corresponding plasma of ST-segment elevation myocardial infarction (STEMI) patients. Network analysis revealed 3 proteins either reduced (F5; FN1) or absent (CLEC3B) in the PR of STEMI patients that are strongly connected to both the clotting cascade and major druggable targets on platelets. This moderated proteomic signature may prove useful for non-invasive risk assessment of the progression of coronary artery disease. These data further contribute to the growing evidence-base of using the platelet releasate as a predictor of pathological state and disease severity.

## Introduction

Circulatory platelets are primary responders at the site of vascular or tissue injury and are active accomplices in vascular inflammation, atherothrombotic progression and thromboischemic diseases (1–3). With an enriched source of effector proteins stored in their granule repertoire (4, 5), they can contribute significantly to circulating levels of pro/anti-inflammatory mediators by releasing their powerful cocktail of soluble, cleaved and vesicular factors, termed the “platelet releasate” (PR), at the site of injury (4–8). This stockpile of platelet cargo, with both pathological and regenerative potential that can direct the course of thromboinflammation (9), provides a directly accessible discrete set of proteins for non-invasive biomarker analysis, possibly to predict cardiovascular outcome among patient groups in near future.

Acute coronary syndromes (ACS) can be categorized as unstable angina, non-ST segment elevation (NSTE) myocardial infarction, and ST-segment elevation myocardial infarction (STEMI). STEMI usually results from prolonged thrombotic occlusion of the coronary artery as thrombus formation ensues following rupture of an atheromatous plaque (10). Platelets circulate in a hyper-reactive state in the circulation of ACS patients. In fact, the number of platelet-derived microparticles (PMPs) is enhanced following acute myocardial infarction and associated with the extent of myocardial damage (11). Furthermore, circulatory platelets release chemokines such as CXCL12, CXCL16, levels of which are elevated in the plasma of ACS patients (12). Thus, following an ischemic episode anti-platelet therapies are recommended to keep subsequent hyper-coagulatory and thrombotic activities in check, and to prevent future ischemic events in these patients.

Despite previously recognized importance of platelets and platelet derived factors in CAD (13–15), proteomic studies investigating the PR have not been performed. We recently established that the PR is highly reproducible across 32 healthy adults, with low variation in secretion levels for individual PR proteins (16). As there is accumulating evidence that circulating platelets may sense or be “educated” by their environment (17, 18); we hypothesized that the PR may alter between stable angina pectoris (SAP) and STEMI and that such alterations may be relevant to disease progression. To address this, we performed comparative label-free quantitative (LFQ) proteomic profiling of the platelet releasate (PR), from STEMI vs. SAP patients using a tandem mass spectrometry (MS) approach.

## Materials and Methods

### Patient Characteristics and Blood Sampling

Blood samples were collected during PCI from the from the TUEPIC clinical registry of coronary artery disease (CAD) patients. All subjects gave written informed consent in accordance with the Declaration of Helsinki. Patients were admitted and treated at the department of Cardiology and Cardiovascular Diseases at the University Clinic of Tübingen, Germany. We included consecutive patients with symptomatic CAD (stable angina pectoris-SAP *n* = 14, ST-elevation myocardial infarction -STEMI *n* = 13). The study was approved by the institutional ethics committee (270/2011BO1) and complies with the declaration of Helsinki and the good clinical practice guidelines (19). Baseline characteristics of patients are tabulated in Table 1. All STEMI patients were directly transferred to the catheterization laboratory for direct percutaneous coronary intervention (PCI). Patients with STEMI were treated with 500 mg aspirin and 5,000 U of non-fractionated heparin at time of diagnosis of STEMI. For platelet analysis whole blood was taken at the beginning of the catherization procedure from the inserted 6 french arterial sheet before additional antithrombotic therapy was given (ACT-adjusted heparin and ticagrelor). Further, in patients with SAP whole blood was collected from the arterial access site before planned PCI after administration of 500 mg aspirin and 5,000 U of non-fractionated heparin. Thereafter, dual antiplatelet therapy was initiated with a loading dose of clopidogrel (20).

**Table 1:** Patient demographics, cardiovascular risk factors, medication on admission and lipid profile parameters in those with STEMI and SAP.

**Characteristics**	**All (*n =* 27)**	**STEMI (*n =* 13)**	**SAP (*n =* 14)**	***p*-value**
Male *n* (%)	21 (77.8%)	11 (84.6%)	10 (71.4%)	0.678
Age, years (mean ± SD)	67.81 (±12.9)	71.46 (± 11.8)	64.42 (±13.5)	0.163
Body mass index (mean ± SD)	27.31 (±4.0)	25.23 (±2.6)	28.44 (±4.3)	0.116
**Cardiovascular risk factors**				
Arterial Hypertension *n* (%)	22 (81.5%)	11 (84.6%)	11 (78.6%)	0.686
Hyperlipidemia *n* (%)	11 (40.7%)	5 (38.5%)	6 (42.9%)	0.082
Diabetes mellitus *n* (%)	8 (29.6%)	3 (23.1%)	5 (35.7%)	0.472
Current smoking *n* (%)	6 (22.2%)	2 (15.4%)	4 (28.6%)	0.410
Obesity *n* (%)	6 (22.2%)	1 (7.7%)	5 (35.7%)	0.080
Renal function (GFR) (Mean ± SD)	79.26 (±24.7)	80.86 (±26.8)	77.66 (±23.3)	0.748
**Medication on admission**				
Acetylsalicylic acid *n* (%)	11 (40.7%)	2 (15.4%)	9 (64.3%)	0.017
Clopidogrel n (%)	4 (14.81%)	0 (0.0%)	4 (28.6%)	0.011
Prasugrel n (%)	1 (3.7%)	0 (0.0%)	1 (7.1%)	0.024
Ticagrelor *n* (%)	1 (3.7%)	0 (0.0%)	1 (7.1%)	0.024
Oral anticoagulants *n* (%)	3 (11.1%)	0 (0.0%)	3 (21.4%)	0.014
Angiotensin-converting enzyme inhibitors *n* (%)	8 (29.6%)	1 (7.7%)	7 (50.0%)	0.011
Angiotensin II receptor antagonists *n* (%)	4 (14.8%)	2 (15.4%)	2 (14.3%)	0.372
Beta-blockers *n* (%)	13 (48.1%)	3 (23.1%)	10 (71.4%)	0.016
Statins *n* (%)	10 (37.0%)	1 (7.7%)	9 (64.3%)	0.004
**Lipid profile parameters**				
Total cholesterol mg/dl (mean ± SD)mmol/l (mean ± SD)	167.7 (±33.7) 4.4 (±0.9)	169.0 (±37.0) 4.4 (±1.0)	166.6 (±32.3) 4.3 (±0.8)	0.872
LDL-cholesterol mg/dl (mean ± SD)mmol/l (mean ± SD)	103.3 (±32.1) 2.7 (±0.8)	106.9 (±39.1) 2.8 (±1.0)	101.2 (±28.9) 2.6 (±0.7)	0.721
HDL-cholesterolmg/dl (mean ± SD)mmol/l (mean ± SD)	44.1 (±15.1) 1.1 (±0.4)	39.3 (±21.2) 1.1 (±0.5)	46.0 (±10.3) 1.2 (±0.3)	0.324
Triglyceridesmg/dl (mean ± SD)mmol/l (mean ± SD)	138.8 (±67.0) 1.6 (±0.8)	123.0 (±70.7) 1.4 (±0.8)	152.0 (±63.7) 1.7 (±0.7)	0.306

### Platelet Releasate Isolation

Platelet isolation was performed as we described previously using a series of well-documented serial centrifugations (7, 16, 21–23). In brief, platelet rich plasma was isolated from whole blood by centrifugation (200 × g for 10 min at room temperature [RT]). Platelet rich plasma was then supplemented with 1 μM prostaglandin E1 and remaining erythrocyte contamination was minimized by further centrifugation (150 × g for 7 min at RT). Platelets were then isolated from plasma by centrifugation (600 × g for 10 min at RT) and subsequently washed using a modified Tyrodes buffer followed again by centrifugation (600 × g for 10 min at RT). Platelets were resuspended at 1 x 10^9^ platelets/ml, left to rest for 30 min at RT and then activated with 1 U/ml thrombin (Roche, Basel, Switzerland) under constant stirring for 5 min at 37°C using a Chronolog-700 platelet aggregometer (Chronolog Cor, Manchester, UK). A minimum measured aggregation threshold of 80% was set for patient inclusion in proteomic analysis. Platelet activation was terminated by immediately placing the tube on ice and with the addition of 1 μM prostaglandin E1. Intact platelets and the platelet clot were carefully removed by centrifuging sequentially x3 at 1000 × g for 10 min at 4°C (in the presence of 2% protease and 2% phosphatase inhibitors; Roche) and harvesting the supernatant each time. The final spin yielded the activated PR as before (7, 16, 21, 22), which was stored at −80 °C until further use.

### Mass Spectrometry (MS) Experimental Design and Tandem MS (MS/MS)

PR samples were prepared for MS as we described previously (16). In brief, samples were individually solubilised in RIPA buffer and proteins precipitated overnight with 95 % acetone (4:1 acetone: sample volume) at −20°C. Dried protein pellets were resuspended in 8 M urea/24 mM Tris-HCL, pH 8.2, at 37°C for 1 h. Disulphide bonds were reduced with 5 mM DTT and protected with 15 mM iodoacetamide. PR samples were digested with Lys-C (1:100; Promega, Madison, WI) followed by digestion with trypsin (1:100; Promega). Peptides were purified using ZipTipC18 pipette tips (Millipore, Billerica, MA, USA) and resuspended in 1 % formic acid. Each biological sample was analyzed using a Thermo-Scientific Q-Exactive mass spectrometer connected to a Dionex Ultimate 3,000 (RSLCnano) liquid chromatography (LC) system as described (23). In brief, each biological sample was individually loaded onto a fused silica emitter (75 μm ID), pulled using a laser puller (Sutter Instruments P2000, Novato, CA, USA), packed with Reprocil Pur (Dr. Maisch, Ammerbuch-Entringen, Germany) C18 (1.9 μm; 12 cm in length) reverse-phase media and separated by an increasing acetonitrile gradient over 47 min (flow rate = 250 nL/min) direct into a Q-Exactive MS. The MS was operated in positive ion mode with a capillary temperature of 320°C, and with a potential of 2,300 V applied to the frit. All data was acquired while operating in automatic data-dependent switching mode. A high resolution (70,000) MS scan (300–1,600 m/z) was performed using the Q-Exactive to select the 12 most intense ions prior to MS/MS analysis using high-energy collision dissociation (HCD).

### Protein ID, Quantification, and Statistical Rationale

Raw MS files were analyzed by MaxQuant (MQ) version 1.5.0.30. MS/MS spectra were searched by the Andromeda search engine against a human FASTA (August 2016) obtained from UniProt. Raw MS data has been deposited to the ProteomeXchange Consortium via the PRIDE partner repository with the dataset identifier PXD009356. MQ analysis included an initial search with a precursor mass tolerance of 20 ppm the results of which were used for mass recalibration. In the main Andromeda search precursor mass and fragment mass had an initial mass tolerance of 6 and 20 ppm, respectively. The search included fixed modification of carbamidomethyl cysteine. Minimal peptide length was set to 7 amino acids and a maximum of 2 miscleavages was allowed. The false discovery rate (FDR) was set to 0.01 for peptide/protein identification. For quantitative comparison between samples we used label-free quantification (LFQ) with a minimum of two ratio counts to determine the normalized protein intensity. LFQ intensities were assigned to identified proteins by comparing the area under the curve of the signal intensity for any given peptide as we performed previously (23).

Data was processed in the Perseus open framework (http://www.perseus-framework.org). Protein IDs were filtered to eliminate identifications from the reverse database, proteins only identified by site, and common contaminants. LFQ intensity values were Log2 transformed. A protein was included if it was identified in at least 50% of samples in at least one (SAP or STEMI) patient cohort. Panther (v13; released 2017-11-12; http://pantherdb.org/) was used to assign gene ontology (GO) with Bonferroni correction against the 2017_04 release of ReferenceProteome dataset. STRING (http://string-db.org/; v10.5) was used to allocate high confidence (minimum required interaction score of 0.700) protein interactions utilizing experimental and database sources.

**Figure 1:**
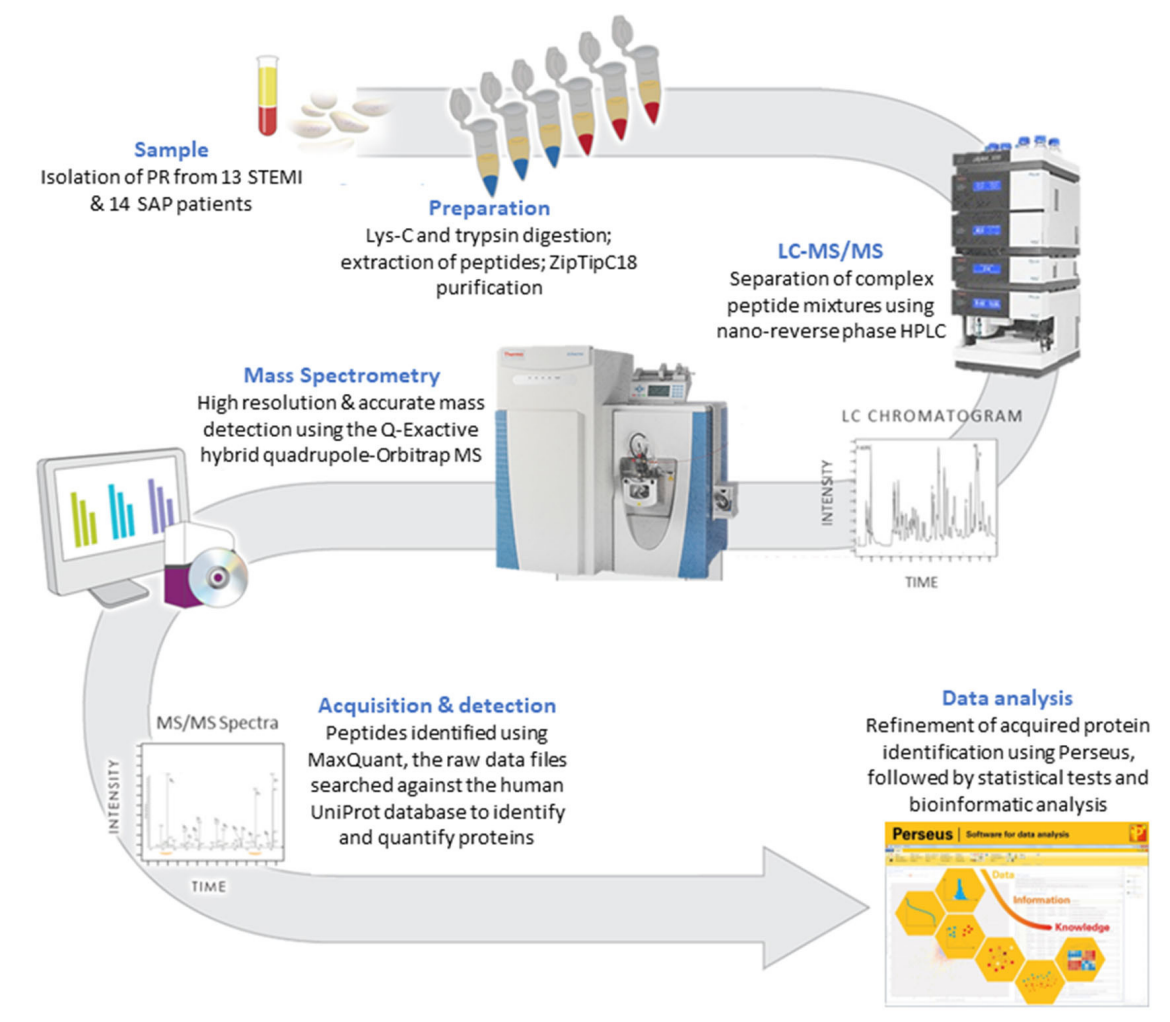
A flow chart of the methodology used to compare the PR proteome in STEMI vs. SAP.

A methodology flow chart can be found in Figure 1.

## Results

**Figure 2:**
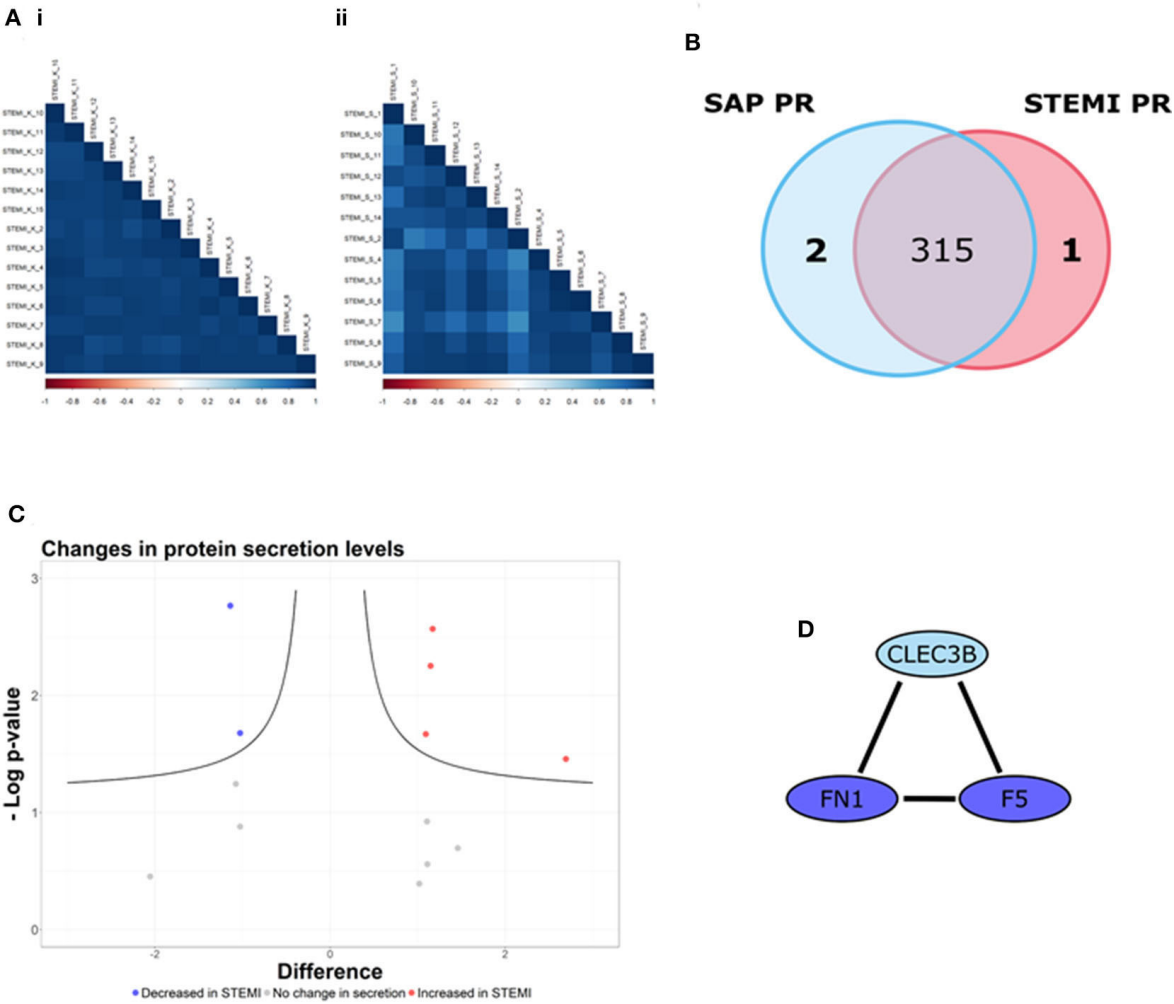
The PR is different between STEMI and SAP. **(A)** Strong inter-patient correlations (*r* > 0.88) were found in the 13 STEMI and 14 SAP platelet releasate (PR) samples. **(B)** 318 PR proteins were identified (in at least 50% of samples in at least one patient cohort), including one PR protein unique to STEMI (red) and two PR proteins unique to SAP (blue). **(C)** Volcano plot of STEMI vs. SAP PR proteomes (x-axis representing the difference between the mean log2 LFQ values of STEMI to SAP PR; y-axis, the negative log transformed *p*-value) representing the proteins significantly altered in each condition. The black hyperbolic curved lines show the threshold for statistical significance, where a false discovery rate of 0.05 and an S_0_ (minimal difference between the mean log2 LFQ values) of 0.1 was used. Of the 14 proteins used for statistical comparison (difference between the mean log2 LFQ values >1/1), our analysis revealed that the secretion levels of 4 proteins were significantly increased in STEMI PR (red dots) whereas 2 proteins were significantly decreased in STEMI PR (blue dots). The secretion levels of the remaining 8 proteins were unchanged (gray dots). **(D)** The STRING database (http://string-db.org/; version 10.5) was used to allocate protein interactions utilizing experimental and database sources. An interaction network of high confidence (>/= 0.700 denoted by line thickness) consisting of three proteins (F5; FN1; CLEC3B) was revealed.

Here, we used LFQ-proteomic profiling to compare the PR proteome of SAP and STEMI (Figure 1). In brief, 1 U/ml thrombin-activated PR was isolated from 13 STEMI and from 14 SAP patients and analyzed by LC-MS/MS (Figure 1). Utilizing statistical algorithms within the Perseus open framework, we examined the biological reproducibility of the LFQ-proteomic analysis between patients in each group using Pearson correlation coefficient analysis (*r*) (Figure 2A). Strong inter-patient reproducibility was observed across our PR samples, averaging at *r* = 0.938 ± 0.023 for SAP patients (Figure 2Ai; Supplementary Table 1) and *r* = 0.878 ± 0.088 for STEMI patients (Figure 2Aii; Supplementary Table 2).

318 PR proteins were identified (in at least 50% of samples in at least one patient cohort) including 2 proteins tetranectin (CLEC3B) and heat shock protein 90-alpha (HSP90AA1) uniquely released from SAP platelets and 1 protein fumarylacetoacetase (FAH) found released only from STEMI platelets (Figure 2B). Supplementary Table 3 displays the log2 transformed LFQ intensity values for the 318 proteins in each sample. Using Perseus software, we determined if there were differences in the secretion levels of the 315 shared proteins. The secretion levels of 301/315 (96%) proteins were deemed unchanged (difference between the mean log2 LFQ values −1/1) and thus these proteins were excluded from further statistical analysis (24). The alteration in the secretion levels of the remaining 14 proteins (difference between the mean log2 LFQ values >1/1) were evaluated for statistical significance (*p* < 0.05) utilizing Student's *t*-test analysis with an FDR value of 0.05 and an S_0_ correction factor of 0.1, denoted by the black hyperbolic lines (Figure 2C) (24). We found that the secretion levels of 4 proteins (red dots; Figure 2C) were significantly increased in STEMI patients in comparison to SAP patients namely glucose-6-phosphate isomerase (GPI), protein disulfide-isomerase A3 (PDIA3), serum amyloid A-1 protein (SAA1) and superoxide dismutase (SOD1). Moreover, the secretion levels of a further 2 proteins (blue dots; Figure 2C) were significantly reduced in STEMI patients when compared to SAP PR, specifically coagulation factor V (F5) and fibronectin (FN1). The secretion levels of the remaining 8 proteins were not significantly changed (gray dots).

GO biological pathway analysis (Supplementary Table 4) was performed on the 9 differential proteins (For a list see Table 2) and revealed a significant enrichment of functional terms including “platelet degranulation” (GO:0002576) (4 proteins: SOD1; FN1; CLEC3B; F5) and “regulated exocytosis” (GO:0045055) (6 proteins: HSP90AA1; SOD1; GPI; FN1; CLEC3B; F5). Strikingly, all 9 differential proteins (HSP90AA1; SAA1; SOD1; GPI; PDIA3; FN1; CLEC3B; F5; FAH) were associated with “vesicle” and “extracellular vesicle” when GO cellular component was analyzed (Supplementary Table 5). Using nanoparticle tracking analysis (NTA), we detected particles in the exosomal-vesicle size range (30–120 nm in size) in the available PR and corresponding plasma (25) from our two patient groups (Supplementary Methods; Supplementary Figures 1A,B). While it is important to note that NTA cannot distinguish between small EVs and lipoproteins, total particle counts (0–200 nm) did reveal a significant decrease in circulating smaller particles (<200 nm) in the corresponding plasma of STEMI vs. SAP patients (*p* = 0.048) (Supplementary Figure 1B).

**Table 2:** 9 PR proteins differentially released in STEMI vs. SAP.

**Gene name**	**Protein name**	**SAP log_**2**_ LFQ**	**STEMI log_**2**_ LFQ**	**Difference (mean log_**2**_ LFQ values)**	**Function**
**Proteins unique to STEMI**					
FAH	Fumarylacetoacetase	NaN	22.398	NaN	Final enzyme in the tyrosine catabolism pathway. FAH deficiency is associated with Type 1 hereditary tyrosinemia
**Proteins unique to SAP**					
CLEC3B	Tetranectin	24.613	NaN	NaN	Binds to Kringle 4 domain of plasminogen. May be involved in the packaging of molecules destined for exocytosis.
HSP90AA1	Heat shock protein HSP 90-alpha	24.132	NaN	NaN	Molecular chaperone, involved in platelet activation. Extracellularly, mediates immune-regulatory & tissue remodeling.
**Proteins with increased secretion in STEMI**					
GPI	Glucose-6-phosphate isomerase	24.76	26.013	1.171	Glycolytic enzyme. Can also function extracellularly as a cytokine, an angiogenic, or neurotrophic factor.
PDIA3	Protein disulfide-isomerase A3	26.026	26.671	1.093	Protein folding, Molecular chaperone, secreted from platelets, Mediates platelet aggregation, haemostasis & thrombosis.
SAA1	Serum amyloid A-1 protein	22.977	23.784	2.692	Major acute phase protein highly expressed in inflammation/tissue injury. Plays a role in HDL metabolism & cholesterol homeostasis. High levels associated with chronic inflammatory diseases including atherosclerosis.
SOD1	Superoxide dismutase	25.693	27.181	1.147	One of two isozymes responsible for destroying free superoxide radicals in the body
**Proteins with decreased secretion in STEMI**					
F5	Coagulation factor V	28.229	27.63	1.679	Critical cofactor for the prothrombinase activity of Factor Xa that results in the activation of prothrombin to thrombin
FN1	Fibronectin	29.416	28.669	2.768	Component of subendothelial matrices and abundant in plasma. Important for stabilization of platelet aggregates after vascular injury.

*Nine proteins were found to be differentially released in the PR of STEMI vs. SAP. Associated gene name, protein name, the average log_2_ LFQ in both SAP and STEMI, the difference between the mean log_2_ LFQ values and the relevant known function of each protein are given*.

These 9 differentially secreted proteins were also subjected to a network analysis using the STRING database (v10.5) (Figure 2D; Supplementary Figure 1C). This revealed an interaction network of high confidence of three α-granular proteins coagulation factor V (F5), fibronectin (FN1) (26) and tetranectin (CLEC3B) (Figure 2D) (27). Interestingly, the secretion levels of the three proteins in this network were either significantly reduced (F5; FN1) or absent (CLEC3B) in the PR of our STEMI patients (Table 2). Secondary protein interactions of these differential proteins highlighted the close connection with the clotting cascade (F2; F10) as well as the major platelet integrins β_3_ (ITGB3) and α_V_ (ITGAV) (Supplementary Figure 1C).

## Discussion

A wealth of information has been gleaned on the PR from platelet proteomic studies, especially over the last few years with dramatic improvements in MS-based proteomics technology (5, 7, 16, 28, 29). Here, we utilized recent advances in label-free quantitation (30) together with more sensitive MS instrumentation to demonstrate the exclusive presence and differential abundance of specific proteomic signatures in the PR from SAP and STEMI patients. Three hundred and eighteen PR proteins were identified, with 9 proteins differentially released. Strikingly, all 9 differential proteins were associated with the GO cellular component term “extracellular vesicle” and we detected reduced levels of smaller particles, which include small EVs (<200 nm), in plasma of STEMI patients. We did not measure larger EVs in our samples, which was an oversight, however these smaller EVs (exosomes) can provide a delivery vehicle for intercellular communication. Whether these smaller EVs mediate protective or pathogenic effects in cardiovascular disease has however yet to be determined (31).

Five proteins were observed to be either exclusively found [fumarylacetoacetase (FAH)] or have increased expression in [glucose-6-phosphate isomerase (GPI), protein disulfide-isomerase A3 (PDIA3), serum amyloid A-1 protein (SAA1) and superoxide dismutase (SOD1)] the PR of STEMI patients on admission in comparison to SAP. FAH is an enzyme involved in the tyrosine catabolism pathway whilst GPI functions in glycolysis. While the pathophysiological significance of increased circulating FAH is unknown, GPI can function extracellularly as a tumor-secreted cytokine or as an angiogenic factor that stimulates endothelial cell motility (32). Although PDIA3 is involved in protein folding in the endoplasmic reticulum, platelets have been shown to release PDIA3 in response to vascular injury, where it can drive platelet activation and subsequent thrombus formation. In fact, PDIA3 is incorporated into the developing thrombus and inhibition can reduce arterial thrombus formation (33, 34). SAA1 is a major acute phase protein that is highly expressed in response to inflammation and tissue injury. Our observation of increased SAA1 in STEMI is consistent with previous reports (35) where high levels of SAA1 can be predictive of coronary artery disease and cardiovascular outcome (36). Therefore, enhanced secretion of GPI, PDIA3 and SAA1 in the PR from STEMI patients with acute thromboischemic events is in accordance with their assigned functions. However, while higher concentrations of circulating SOD1 (one of two isozymes responsible for destroying free superoxide radicals) have been found in STEMI when compared to healthy controls, there is conflicting evidence as to whether levels may vary significantly in comparison to SAP (37).

A further 4 proteins were found to be either absent from [tetranectin (CLEC3B); heat shock protein 90-alpha (HSP90AA1)], or statistically reduced in [coagulation factor V (F5) and fibronectin (FN1)], the PR of STEMI patients when compared to SAP. Tetranectin (CLEC3B), a member of the C-type lectin domain family with importance in platelet biology (38), has already emerged as a marker of CAD; where levels were reduced in SAP patients in comparison to healthy controls (35, 39). In fact, an incremental reduction in serum levels and concomitant expression in atherosclerotic lesions correlated with disease progression (39). Moreover, CLEC3B induces plasminogen activation (40), which is an important fibrinolytic pathway that may be deficient in the ACS cohort. HSP90AA1 is a molecular chaperone, known to be involved in thrombin-induced platelet activation (41). Extracellularly, HSP90AA1 has been shown to mediate immune-regulatory and tissue remodeling effects (42) and increased HSP90 levels are associated with atherosclerotic plaque instability (43). As CAD severity has also been inversely associated with circulating HSP levels (44), the absence of CLEC3B and HSP90AA1 from the PR of STEMI patients may reflect circulating activated platelets and increased plaque uptake; however, the pathophysiological significance of these particular PR proteins in the peripheral circulation of CAD patients remains to be explored.

The levels of F5 and FN1 were also found decreased in the PR of STEMI patients. Platelet-derived Factor V (F5) was found to be a critical mediator of arterial thrombosis in mice (45) and CAD patients show significantly higher levels of plasma FN1 as compared to healthy subjects, with platelet FN1 levels correlating with severity of disease (46, 47). Decreased F5 and FN1 levels in PR of STEMI patients as compared to SAP patients may reflect possible consumption of these proteins during acute MI. Together with CLEC3B, F5, and FN1 emerged as a modified network in the PR of STEMI patients. This triad is strongly connected to both the clotting cascade (association with F2; F10) and major integrin receptors on platelets (association with ITGB3 and ITGAV) that have emerged in previous proteomics studies of platelets in ACS (48), highlighting these proteins as relevant players in the acute event.

In conclusion, our comprehensive analysis of the PR proteome in STEMI provides a moderated protein network which may be useful for non-invasive risk assessment of the onset and progression of coronary artery disease.

## Data Availability Statement

Raw MS data has been deposited to the ProteomeXchange Consortium via the PRIDE partner repository with the dataset identifier PXD009356.

## Ethics Statement

The studies involving human participants were reviewed and approved by Ethik-Kommission an der Medizinischen Fakultät der Eberhard-Karls-Universität und am Universitätsklinikum Tübingen. The patients/participants provided their written informed consent to participate in this study.

## Author Contributions

PBM, MP, PS, MZ, PM, MC, KW, FN, and MG designed research. MP, PS, MZ, PM, KW, and MH performed the experiments. PBM, MP, PS, MZ, PM, MC, DR, FN, and MG analyzed the data. PBM, PS, MC, DR, SC, FN, and MG wrote the paper.

## Conflict of Interest

The authors declare that the research was conducted in the absence of any commercial or financial relationships that could be construed as a potential conflict of interest.

## Abbreviations

ACS: Acute coronary syndrome
SAP: stable angina pectoris
STEMI: ST-segment elevation myocardial infarction
PR: platelet releasate
LFQ: label-free quantification
LC-MS/MS: liquid chromatography tandem mass spectrometry
MQ: MaxQuant
CV: coefficient of variation
GO: Gene Ontology.
