# Supplementary figures and images for: Comparative Platelet Releasate Proteomic Profiling of Acute Coronary Syndrome versus Stable Coronary Artery Disease
Source: Front Cardiovasc Med. 2020 Jun 24;7:101. doi: 10.3389/fcvm.2020.00101 (PMC7328343; doi:10.3389/fcvm.2020.00101)

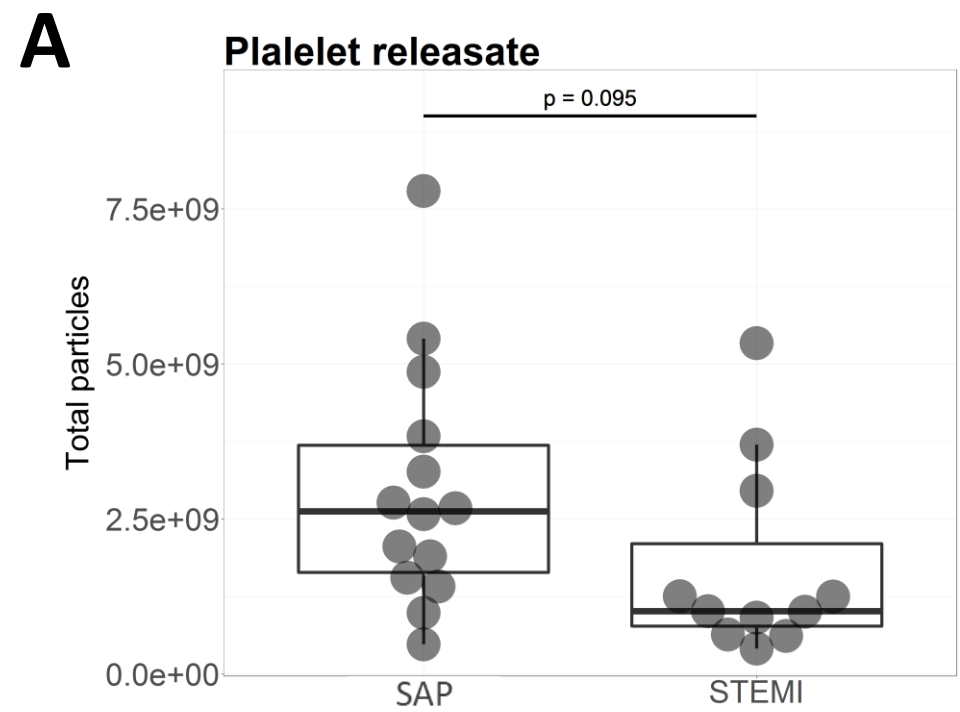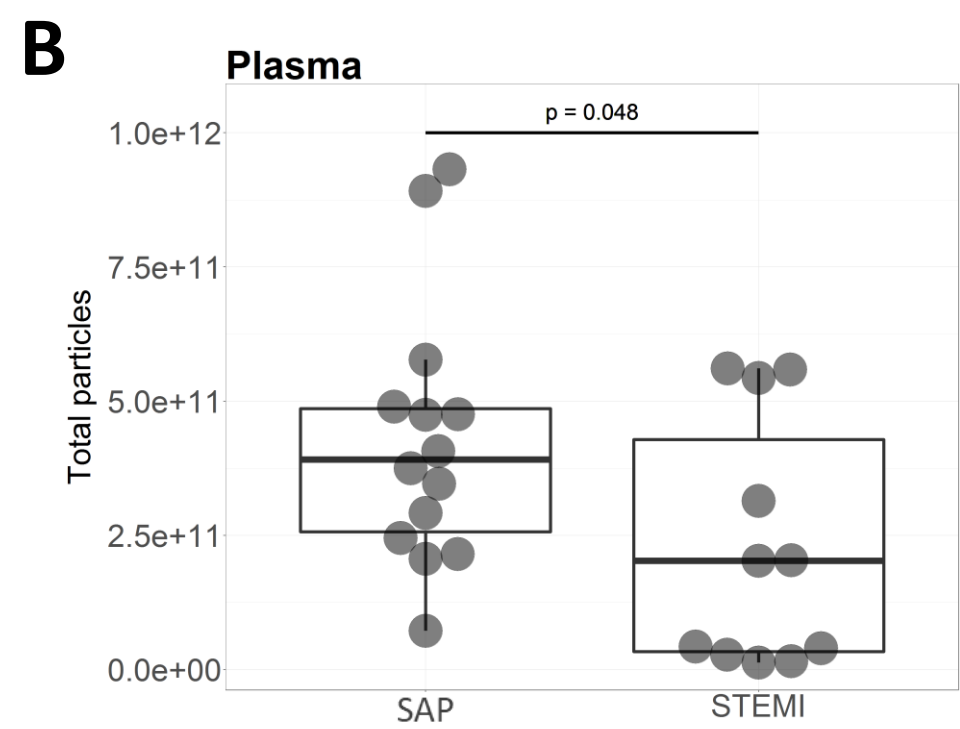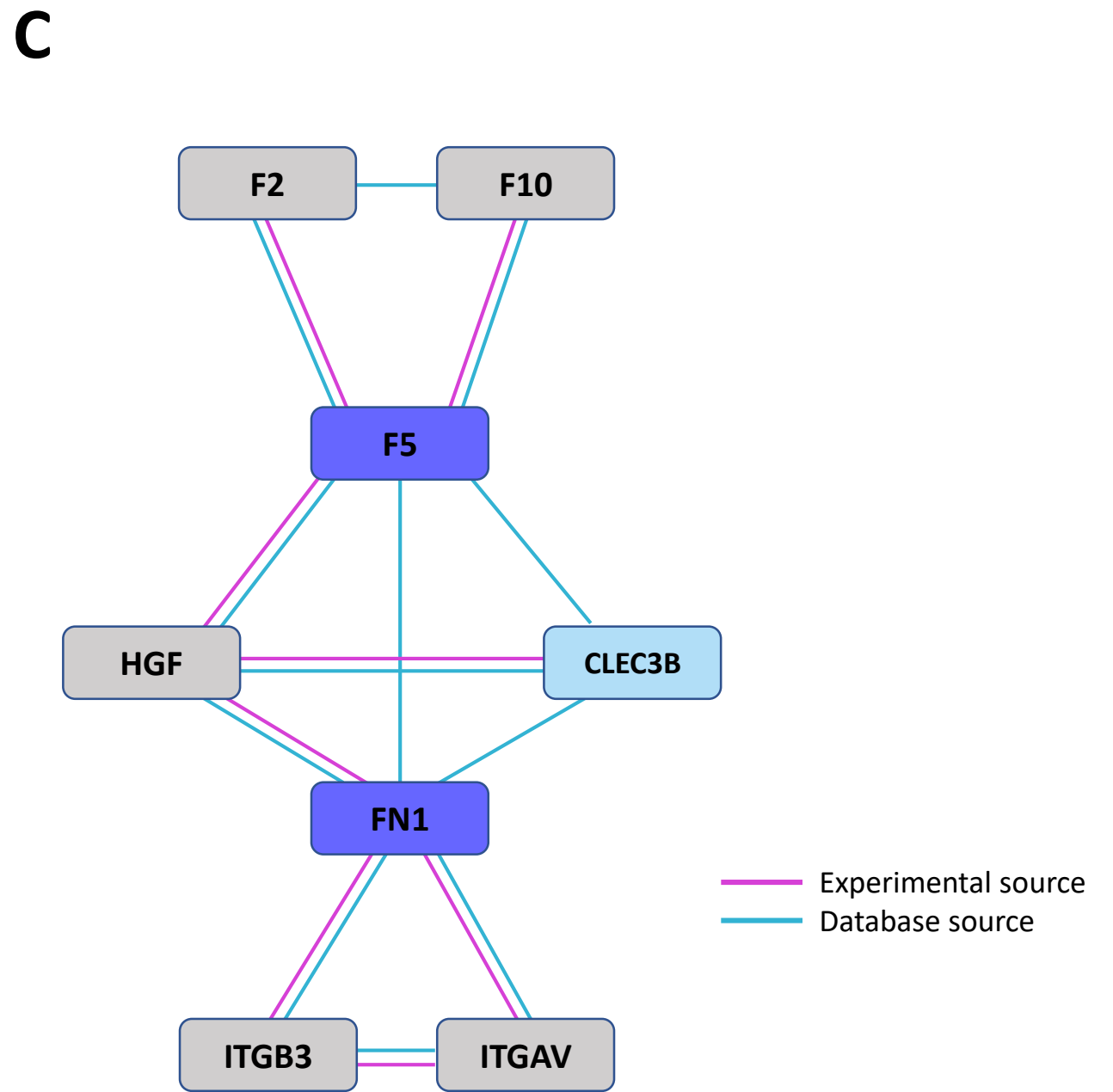

**SUPPLEMENTARY FIGURE 1**

Supplement: Supplementary file 6 [file Image_1.pdf]
