# Supplementary material for: Comparative Platelet Releasate Proteomic Profiling of Acute Coronary Syndrome versus Stable Coronary Artery Disease
Source: Front Cardiovasc Med. 2020 Jun 24;7:101. doi: 10.3389/fcvm.2020.00101 (PMC7328343; doi:10.3389/fcvm.2020.00101)
Supplement: Supplementary file 7 [file Data_Sheet_1.docx]

**SUPPLEMENTARY DATA**

**Supplementary Experimental Procedures:**

**LC-MS/MS:**

Protein concentration was assessed by Bradford protein assay (Bio-Rad, Hercules, CA, USA) using bovine serum albumin (BSA) (Sigma-Aldrich) as standard for the calibration curve and for each sample 30 µg proteins were precipitated with 95 % acetone (4:1 acetone:sample volume) overnight. Dried protein pellets were resuspended in 8 M Urea/ 25 mM Tris-HCL, pH 8.2, at 37 ^o^C with gentle agitation. Disulphide bonds were reduced with 5 mM DTT and protected with 15 mM iodoacetamide. Proteins were first digested with Lys-C (1:100; Promega, Madison, WI) followed by digestion with trypsin (1:100; Promega). Peptides were purified using ZipTipC18 pipette tips according to manufacturer instructions (Millipore, Billerica, MA, USA) and resuspended in 1 % formic acid. 5 µg of each sample was used for tandem MS run.

Each sample was run on a Thermo Scientific Q Exactive mass spectrometer connected to a Dionex Ultimate 3000 (RSLCnano) chromatography system. Tryptic peptides were resuspended in 0.1% formic acid. Each sample was loaded onto a fused silica emitter (75μm ID), pulled using a laser puller (Sutter Instruments P2000, Novato, CA, USA), packed with Reprocil Pur (Dr Maisch, Ammerbuch-Entringen, Germany) C18 (1.9μm; 12 cm in length) reverse phase media and were separated by an increasing acetonitrile gradient over 47 minutes at a flow rate of 250 nL/min direct into a Q-Exactive MS. The MS was operated in positive ion mode with a capillary temperature of 320 °C, and with a potential of 2300 V applied to the frit. All data was acquired while operating in automatic data dependent switching mode. A high resolution (70,000) MS scan (300-1600 m/z) was performed using the Q Exactive to select the 12 most intense ions prior to MS/MS analysis using high-energy collision dissociation (HCD).

**Nanoparticle tracking analysis:**

Plasma and platelet releasate particle size distribution was determined for the corresponding and available patient samples (11 STEMI and all 14 SAP) by nanoparticle tracking analysis (NTA) using a Malvern NanoSight NS300 (Malvern Technologies, Malvern, UK) with a high-sensitivity sCMOS camera and a 488 nm laser. Plasma was diluted 1:100 - 1:1000, platelet releasate was diluted 1:20 – 1:100 in particle free PBS (Gibco, Waltham, MA, USA) to an acceptable concentration according to manufacturer’s instructions. Samples were analysed under constant flow conditions (flow rate = 50) at 25°c. 15 x 60 second videos were captured with a camera level of 16. Data was analysed using NTA 3.1.54 software with a detection threshold of 5.

**Supplementary Figure 1**

**
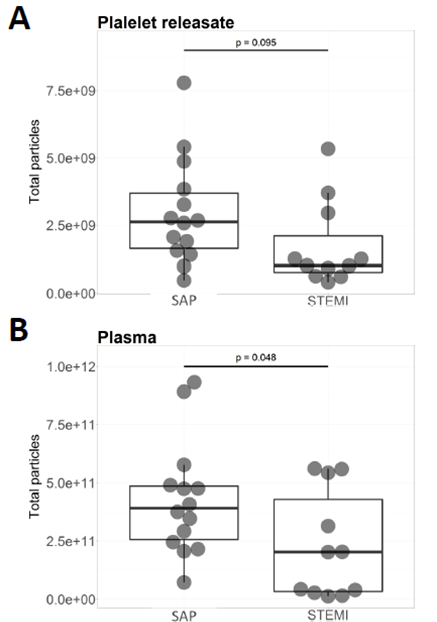
**


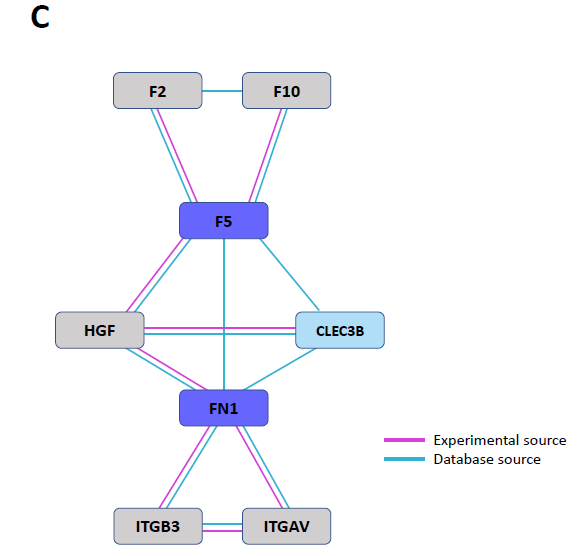


**Supplementary Figure 1:** (A) Total particles in the platelet releasate between 0 – 200nm isolated from STEMI patients (*n*=11) compared to SAP patients (*n*=14). (B) Total particles in plasma between 0 – 200 nm isolated from STEMI patients (*n*=11) compared to SAP patients (*n*=14). (C) An interaction network incorporating secondary interactors (grey) was constructed around the significantly reduced (FN1 & F5; dark blue) and absent (CLEC3B; light blue) proteins in the PR of our STEMI patients, by importing known interactions based on experimental (pink line) and database (blue line) sources from STRING database (v10.5).

**SUPPLEMENTARY TABLE LEGENDS:**

**Supplementary Table 1: Pearson correlation coefficients (*r*) of SAP PR proteome illustrate a very strong biological reproducibility.**

Utilising statistical algorithms within the Perseus open framework, Pearson correlation coefficient analysis (*r*) was performed on all proteins identified in the PR from our 14 SAP (STEMI_K) patients to assess correlation. We found very strong reproducibility, averaging at 0.938±0.023.

**Supplementary Table 2: Pearson correlation coefficients (*r*) of STEMI PR proteome illustrate a strong biological reproducibility.**

Utilising statistical algorithms within the Perseus open framework, Pearson correlation coefficient analysis (*r*) was performed on all proteins identified in the PR from 13 STEMI (STEMI_S) patients to assess correlation. We found strong reproducibility, averaging at 0.878±0.088.

**Supplementary Table 3: 318 proteins identified in total from STEMI and SAP PR.**

27 platelet releasate samples [13 STEMI (STEMI_S) and 14 SAP (STEMI_K) patients] were analysed by LC-MS/MS to reveal a proteomic profile of 318 proteins. Protein IDs were filtered to eliminate identifications from the reverse database, proteins only identified by site, and common contaminants. A protein was only included if it was identified in at least 50% of samples in at least one (STEMI or SAP) group. Protein ID, peptide sequences identified, molecular weight, PEP significance score, MS/MS count, intensity and the log_2_ transformed LFQ intensity values across each MS run are provided for each protein (columns A-AL).

**Supplementary Table 4: GO biological pathway analysis.**

GO biological pathway analysis was performed using Panther (v13.0; ReferenceProteome dataset released 2017_04; http://pantherdb.org/). All 9 differential proteins were annotated for defined biological pathway terms including ‘platelet degranulation’, ‘secretion’ and ‘vesicle-mediated transport’ etc and the proteins attributed to each pathway as well as the corresponding gene names are denoted in the columns labelled "Enriched genes" and "Genes" respectively. Expected, fold enrichment, enrichment and corrected *p*-values are also given and represent the likelihood that the intersection of a given list with any given biological pathway is due to random chance. Bonferroni corrected *p*-values <0.05 indicate a statistically significant, non-random association.

**Supplementary Table 5: GO cellular component analysis.**

GO cellular component analysis was performed using Panther (v13.0; ReferenceProteome dataset released 2017_04; http://pantherdb.org/). All 9 differential proteins were annotated for defined cellular component terms including ‘vesicle’, ‘secretory granule’ ‘secretory vesicle’ and ‘extracellular vesicle’ etc and the proteins attributed to each pathway as well as the corresponding gene names are denoted in the columns labelled "Enriched genes" and "Genes" respectively. Expected, fold enrichment, enrichment and corrected *p*-values are also given and represent the likelihood that the intersection of a given list with any given biological pathway is due to random chance. Bonferroni corrected *p*-values <0.05 indicate a statistically significant, non-random association.
